# Modulation of base excision repair of 8-oxoguanine by the nucleotide sequence

**DOI:** 10.1093/nar/gkt620

**Published:** 2013-07-17

**Authors:** Julia Allgayer, Nataliya Kitsera, Carina von der Lippen, Bernd Epe, Andriy Khobta

**Affiliations:** Institute of Pharmacy and Biochemistry, Johannes Gutenberg University of Mainz, Staudingerweg 5, 55128 Mainz, Germany

## Abstract

8-Oxoguanine (8-oxoG) is a major product of oxidative DNA damage, which induces replication errors and interferes with transcription. By varying the position of single 8-oxoG in a functional gene and manipulating the nucleotide sequence surrounding the lesion, we found that the degree of transcriptional inhibition is independent of the distance from the transcription start or the localization within the transcribed or the non-transcribed DNA strand. However, it is strongly dependent on the sequence context and also proportional to cellular expression of 8-oxoguanine DNA glycosylase (OGG1)—demonstrating that transcriptional arrest does not take place at unrepaired 8-oxoG and proving a causal connection between 8-oxoG excision and the inhibition of transcription. We identified the 5′-CAGGGC[8-oxoG]GACTG-3′ motif as having only minimal transcription-inhibitory potential in cells, based on which we predicted that 8-oxoG excision is particularly inefficient in this sequence context. This anticipation was fully confirmed by direct biochemical assays. Furthermore, in DNA containing a bistranded Cp[8-oxoG]/Cp[8-oxoG] clustered lesion, the excision rates differed between the two strands at least by a factor of 9, clearly demonstrating that the excision preference is defined by the DNA strand asymmetry rather than the overall geometry of the double helix or local duplex stability.

## INTRODUCTION

8-Oxoguanine (8-oxoG) is a more common name for 8-oxo-7,8-dihydroguanine, which is the predominant oxidation product of guanine in genomic DNA. Already under normal physiological conditions, 8-oxoG is generated at a frequency of at least several hundred lesions per human cell per day by reaction of intracellularly produced reactive oxygen species with DNA (1); this rate is further increased under oxidative stress conditions (2,3). Failure of repair mechanisms to properly deal with such a damage load has several detrimental consequences. The first is false pairing of 8-oxoG (in syn-conformation) with adenine, resulting in increased frequency of replication errors (4–6). This lesion-templated misincorporation of dATP by DNA polymerases leads to mutations and cancer, particularly in individuals with mutated MUTYH gene whose product removes adenine from the 8-oxoG/A mispairs (7,8). The second adverse effect of genomic 8-oxoG is erroneous bypass of the lesion by transcribing RNA polymerase II complexes, resulting in RNA mutagenesis and consequent production of aberrant proteins (9). Finally, 8-oxoG causes a decrease in transcriptional output of the damaged gene—so powerful that even a single lesion is sufficient to produce a significant effect (10). Remarkably, transcription is not inhibited in *Ogg1^−^^/^^−^* mouse cells, which are deficient in the base excision repair (BER) of 8-oxoG. These observations led to an idea that BER might interfere with transcription if both processes occur simultaneously (10). Meanwhile, 8-oxoG does not strongly block transcription by RNA polymerase complexes directly encountering the lesion (11–15).

BER of 8-oxoG is initiated by the specific DNA glycosylase OGG1, which is conserved among eukaryotic organisms from yeast to humans (16–21). OGG1 is a bifunctional DNA glycosylase, which performs two distinct enzymatic steps—hydrolysis of the N-glycosidic bond and beta-elimination of the phosphate on the 3′ side of the resulting apurinic (AP) site (22,23). Cells isolated from OGG1-null mice are deficient in repair of 8-oxoG (9,24), and their extracts show no excision of 8-oxoG from double-stranded DNA (12,24,25), strongly suggesting that excision by OGG1 is the major and apparently the only physiologically relevant mechanism of removal of 8-oxoG from nuclear DNA in mammalian cells. In consequence, *Ogg1^−^^/^^−^* mice accumulate significant amounts of 8-oxoG in their organs with age or following the induction of oxidative stress (24–27) and also display increased rates of the characteristic G→T transversion mutations (24).

The presence of OGG1 in wild-type mice and in humans does not fully prevent the 8-oxoG-induced mutagenesis. In particular, high prevalence of somatic G→T transversions in human tumour samples from patients with MUTYH mutations (7) denotes the insufficient repair even in individuals with unaffected OGG1 gene. Because of the limited repair capacity and continuous generation of new DNA damage, significant amounts of 8-oxoG are always present in chromosomal DNA (28). Interestingly, genome-wide distribution of 8-oxoG shows a distinctive non-random pattern (29), thus suggesting a spatial heterogeneity of damage generation and/or repair in cells, the reasons for which are unclear. Here, we used a reporter gene approach to investigate the gene expression in the presence of single 8-oxoG/C base pair, placed in different orientations, in various positions and in different sequence contexts. We found strong variation of the magnitude of the inhibition of the gene expression, which was dependent on the sequence context of 8-oxoG, but not on the other parameters tested. By manipulating the cellular OGG1 levels and the nucleotide sequence surrounding 8-oxoG, we proved that the inhibition of gene expression is caused by excision of 8-oxoG by human OGG1. We further showed that local nucleotide sequence significantly modulates the excision rate of 8-oxoG and, by this means, also transcription of the damaged gene in cells.

## MATERIALS AND METHODS

### Cell lines, enzymes, oligonucleotides

Stable knockdown of OGG1 in HeLa cells is described in Supplementary Information. HeLa cells stably overexpressing human OGG1 protein fused to GFP (30) were obtained from Pablo Radicella (CEA, Fontenaix au Roses). OGG1 protein was obtained either from the Radicella laboratory or from NEB GmbH (Frankfurt am Main, Germany). All other enzymes were either from NEB or from Thermo Scientific (St Leon-Rot, Germany). Synthetic oligonucleotides were all purified by high-performance liquid chromatography and verified by mass-spectrometry. Oligonucleotides to the protein-coding sequence (31) were obtained from Thomas Carell (LMU Munich). All other modified oligonucleotides were purchased from BioSpring GmbH (Frankfurt am Main, Germany).

### Site-specific incorporation of 8-oxoG into vector DNA

Construction of vectors for site-specific incorporation of 8-oxoG into the 5′- or the 3′-untranslated region (UTR) of the enhanced green fluorescent protein (EGFP) gene is described in Supplementary Information, and the sequences are available in Supplementary Figure S1. The desired DNA strand was incised at two specific sites by one of the nicking endonucleases (Nb.Bpu10I, Nt.Bpu10I or Nb.BsrDI), and matching synthetic oligonucleotides (unmodified or containing single 8-oxoG in the specified position) were incorporated by the direct strand exchange procedure. For the protein-coding gene region, we followed the method described previously (10,31), but with a different vector (pZAJ-3w-AGC). For each position of 8-oxoG in the untranslated gene regions, individual vectors were designed and a different nicking endonuclease (Nb.BsrDI) was used, but the strand exchange procedure remained unchanged. The oligonucleotide sequences with references to the corresponding vectors are listed in Supplementary Table S1. The presence of 8-oxoG in vector DNA was always verified by FaPy DNA glycosylase (Fpg) incision assay, as described previously (31).

### Transfections and analyses of gene expression

HeLa cells were co-transfected with equal amounts of one of the EGFP-encoding plasmids with an inserted synthetic oligonucleotide (either unmodified or containing single 8-oxoG) and pDsRed-Monomer-N1 vector (Clontech, Saint-Germain-en-Laye, France). Transfections were performed with the help of Effectene (QIAGEN, Hilden, Germany), and cells processed and fixed at the indicated time intervals, as described previously (10). For the time-course analyses, transfected cells were detached at 8 h and split in several portions. One was directly fixed for analyses, whereas others were plated and fixed at specified time intervals. The method for analysis of EGFP expression by flow cytometry was described in detail previously (32), and a quantitative correlation between the cellular EGFP signal and the RNA transcript levels was established (10).

### Excision of single 8-oxoG from plasmid DNA

For the OGG1 excision analyses, plasmid DNA containing single 8-oxoG in the specified positions (100 ng per 15 μl reaction) was incubated with the indicated amounts of human OGG1 and 1 unit endonuclease IV in 10 mM HEPES (pH 7.5), 1 mM EDTA, 200 mM NaCl, 0.1 g/l BSA. Samples were incubated 1 h at 37°C followed by heat-inactivation 20 min at 65°C and directly analysed by agarose gel electrophoresis in the presence of 0.5 mg/l ethidium bromide. Quantification of DNA in the bands was performed by GelDoc™ XR+ Molecular Imager® with Image Lab™ Software (Bio-Rad Laboratories GmbH, Munich, Germany). For the plasmids used in this study, it was determined that nicked DNA produced a 2.4-fold stronger fluorescence signal than the same amount of covalently closed DNA, which was used as the correction factor for calculation of relative abundance of the incised substrate.

Cell extracts for the cleavage assays were obtained as following. Exponentially growing cells (20 million) were harvested on ice in phosphate buffered saline supplemented with protease inhibitors and centrifuged (200*g*, 5 min at 4°C). Cell pellets were resuspended in 0.5 ml of lysis buffer [20 mM Tris–HCl, (pH 8.0); 1 mM EDTA; 250 mM NaCl] supplemented with protease inhibitors (Roche Diagnostics GmbH, Mannheim). Samples kept on ice-water slurry were sonicated using a Bachofer GM 70 HD ultrasonic processor (Bachofer GmbH, Reutlingen, Germany) equipped with a microtip (20% power setting, four cycles of 40 s, duty cycle 0.4). Insoluble material was pelleted by ultracentrifugation (100 000*g*, 45 min at 4°C). Protein concentration in supernatants was determined by Bradford assay. Extracts were aliquoted for single use and stored at *−*80°C. Excision with cell extracts was performed at the same conditions as for OGG1; however, endonuclease IV was omitted.

### Construction of plasmid containing clustered 8-oxoG

Covalently closed circular plasmid DNA containing a Cp[8-oxoG]/Cp[8oxoG] bistranded clustered lesion was constructed by sequential exchange of native fragments of each of the DNA strands for matching synthetic oligonucleotides, each containing single 8-oxoG, as explained in Supplementary Figure S6. Two plasmids containing single 8-oxoG in the alternative orientations and a plasmid without 8-oxoG were produced by the same procedure, using the appropriate combinations of the modified and unmodified synthetic oligonucleotides. Because Fpg cannot efficiently excise both 8-oxoGs at the bistranded clustered lesion, the success of the second strand exchange reaction was verified by inhibition of the final ligation reaction in a control sample containing the non-phosphorylated oligonucleotide, as described previously (31).

### Mapping of the OGG1 excision at the clustered 8-oxoG

OGG1 excision reactions were performed as for single 8-oxoG, but scaled up. Plasmid DNA was isopropanol precipitated, washed with 70% ethanol and dissolved in Tris–HCl (pH 8.0). The excision products were 3′-end labelled with [α-^32^P]-dGTP (PerkinElmer, Rodgau, Germany), whose specific activity was preliminary adjusted to 300 Ci/mmol with cold dGTP. DNA (1 µg) was incubated with 2 units T4 DNA polymerase (37°C, 5 min) in BSA-supplemented NEBuffer 2 containing 100 µM each dATP, dCTP and dTTP, followed by addition of 20 μCi [α-^32^P]-dGTP. Labelling was performed for 5 min and quenched by addition of cold dNTPs and heat-inactivation (80°C, 20 min). Labelled DNA was directly digested with 25 units DpnI (37°C, 1 h). After another cycle of heat-inactivation, sample volumes were adjusted to 0.2 ml with TE-buffer containing 0.1% SDS and extracted with phenol/chloroform/isoamyl alcohol (25:24:1). DNA was ethanol-precipitated and re-dissolved in 35 μl of formamide loading buffer. Labelled fragments were separated by denaturing electrophoresis in a 10% polyacrylamide gel containing 7 M urea and visualized by gel autoradiography and phosphorimaging. Band intensities were determined by publicly available ImageJ 1.46o software (http://imagej.nih.gov/ij/).

## RESULTS

### 8-OxoG inhibits transcription of a reporter gene in a position-independent manner

**Figure 1.**
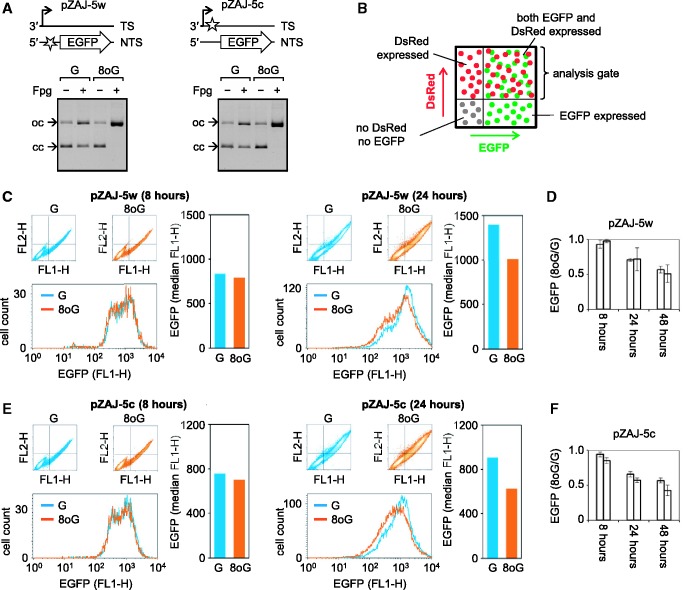
Effect of single 8-oxoG in the 5′-UTR on expression of the EGFP-coding gene in Hela cells. (**A**) Agarose gel analyses of vector DNA harbouring synthetic 18mer oligonucleotides containing either G or 8-oxoG. The aliquots were incubated with Fpg DNA glycosylase to detect 8-oxoG by conversion of covalently closed DNA (cc) to open circles (oc). Diagrams illustrate position of 8-oxoG (star) with respect to transcription start (broken arrow) and the protein-coding DNA sequence (arrow). (**B–F**) Flow cytometric analyses of HeLa cells co-transfected with equal amounts of a vector encoding for DsRed-Monomer and the indicated EGFP encoding plasmids. (B) The principle of quantitative analysis of EGFP expression in transfected cells. (C and E) Results of typical experiments. (D and F) Relative EGFP expression summarized for two independently prepared 8-oxoG/G substrate pairs (separate bars, each representing mean of three transfection experiments±SD). See also Supplementary Figure S2.

We constructed plasmid vectors, suitable for incorporation of synthetic oligonucleotides into the 5′- UTR of the EGFP gene and into the 3′-UTR, by inserting an arbitrarily chosen DNA sequence flanked by two tandemly located sites for the Nb.BsrDI nicking endonuclease. For both locations, the new sequences were placed in alternative orientations (Supplementary Figure S1). Vectors containing the modified 5′-UTR will be further referred to as pZAJ-5w and pZAJ-5c. Similarly, the vectors with modified 3′-UTR were named pZAJ-3w and pZAJ-3c. Tandem Nb.BsrDI sites of each of the four vectors were used to swap the enclosed 18-nt fragment of the native DNA strand for a synthetic deoxyribo-oligonucleotide, according to the principle described previously (31). The oligonucleotide contained single 8-oxoG in a 5′-A[8-oxoG]C context (Supplementary Table S1). Successful incorporation of 8-oxoG was confirmed by analytical incision of the obtained hybrid molecules with Fpg (Figure 1A and Supplementary Figure S2). The vectors obtained by insertion of unmodified oligonucleotides were only partly converted to the open circular form by incision at a small number of Fpg-sensitive sites intrinsically present in the plasmid DNA. In contrast, the vectors harbouring the 8-oxoG containing oligonucleotide were fully incised, thereby indicating that all molecules contained the synthetic 8-oxoG in addition to the basal non-specific DNA damage.

Measurements of EGFP expression in HeLa cells 24 h after transfection with plasmid vectors containing single 8-oxoG in the 5′-UTR of the gene documented a clear decrease of fluorescence, compared with cells transfected with the reference plasmids harbouring the unmodified oligonucleotide (Figure 1B–F). The effect had the same magnitude for 8-oxoG located in the transcribed DNA strand and one in the complementary DNA strand. In cells, fixed 8 h after transfection, the decrease of EGFP expression was not yet observable for both tested positions of 8-oxoG. This result is consistent with the delayed inhibition of transcription reported previously for another construct with a differently positioned 8-oxoG (10) and is useful as an indicator that equal amounts of constructs containing G and 8-oxoG were actually delivered to cells. We also analysed expression of vectors containing single 8-oxoG in the 3′-UTR of the EGFP gene and, as in the 5′-UTR, documented clear inhibitory effects of the lesions on the gene expression, regardless of the DNA strand affected (Supplementary Figure S2). We therefore conclude that single 8-oxoG in the 5′-A[8-oxoG]C context within the transcribed EGFP gene is sufficient to cause inhibition of the gene expression. Within the same DNA sequence context, the inhibition of transcription is not influenced by the distance between 8-oxoG and the transcription start and is independent of the orientation of the affected DNA strand with respect to the direction of transcription.

### The magnitude of inhibition of transcription by 8-oxoG in cells is proportional to the OGG1 expression

**Figure 2.**
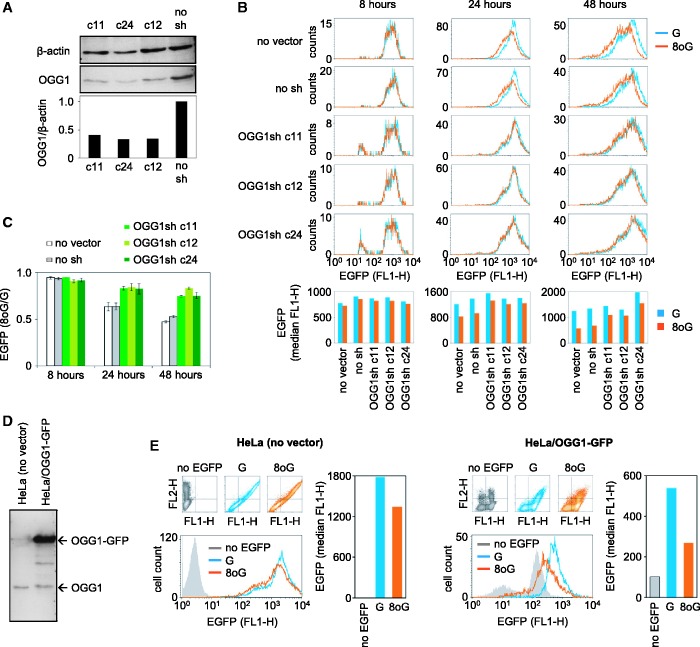
Effect of the OGG1 protein levels in HeLa and the derived cell lines on the expression of vectors containing single 8-oxoG. (**A**) Western blot analysis of OGG1 protein expression in three independent clones expressing OGG1 shRNA and in the clone transfected with the empty vector (no sh). (**B** and **C**) Effects of single 8-oxoG on the EGFP expression in clonal cell lines expressing OGG1 shRNA (OGG1sh) and in the isogenic cell lines with normal OGG1 expression (no vector, no sh). (B) Fluorescence distribution plots and median EGFP fluorescence (below) for a representative transfection experiment. (C) Relative EGFP expression for two independently generated 8-oxoG/G substrate pairs (mean and data range). (**D**) Western blot analysis of cells stably overexpressing the OGG1-GFP fusion protein. (**E**) Effects of the OGG1-GFP overproduction on expression of the plasmid DNA containing single 8-oxoG (24 h post-transfection). Grey represents endogenous fluorescence, which is higher in the OGG1-GFP expressing cells. See also Supplementary Figure S3.

For every investigated position of single 8-oxoG within the EGFP gene, its effects on the gene expression were increasing in magnitude over the time of observation (Figure 1D and F and Supplementary Figure S2). Such a behaviour is in line with a previously proposed indirect mechanism for transcription inhibition by 8-oxoG, according to which not the base modification itself, but a single-strand break generated in result of its excision by OGG1 may produce a transcription-blocking effect (10). We tested this assumption in isogenic HeLa-derived cell lines expressing varying levels of human OGG1. We generated monoclonal cell lines, which stably express an OGG1-specific short hairpin (sh) RNA. The OGG1sh cells show decreased expression levels of the OGG1 protein (Figure 2A) and, accordingly, retarded repair of oxidative base damage (Supplementary Figure S3). The expression of plasmid constructs containing single 8-oxoG in the 5′-UTR of the EGFP gene was significantly improved in cell lines expressing the OGG1 shRNA, compared with a control clone (stably transfected with the empty vector) and with the maternal cell line (Figure 2B and C). The opposite was true for a cell line overexpressing human OGG1, which was previously reported to have a greatly enhanced repair rate for 8-oxoG induced in chromosomal DNA by potassium bromate (30). In this cell line, the inhibition of EGFP expression by single 8-oxoG was enhanced, compared with control cells (Figure 2D and E). Altogether, the results (Figure 2) show that the inhibition of the EGFP gene expression by 8-oxoG is in direct relationship with the amounts of OGG1 and the BER activity in host cells. This finding strongly supports the notion that excision of the lesion by OGG1 provides the underlying mechanism for downregulation of the gene transcription, whereas the unrepaired 8-oxoG does not cause a transcriptional arrest.

### DNA sequence defines the rate of excision of 8-oxoG and thereby the magnitude of inhibition of transcription

**Figure 3.**
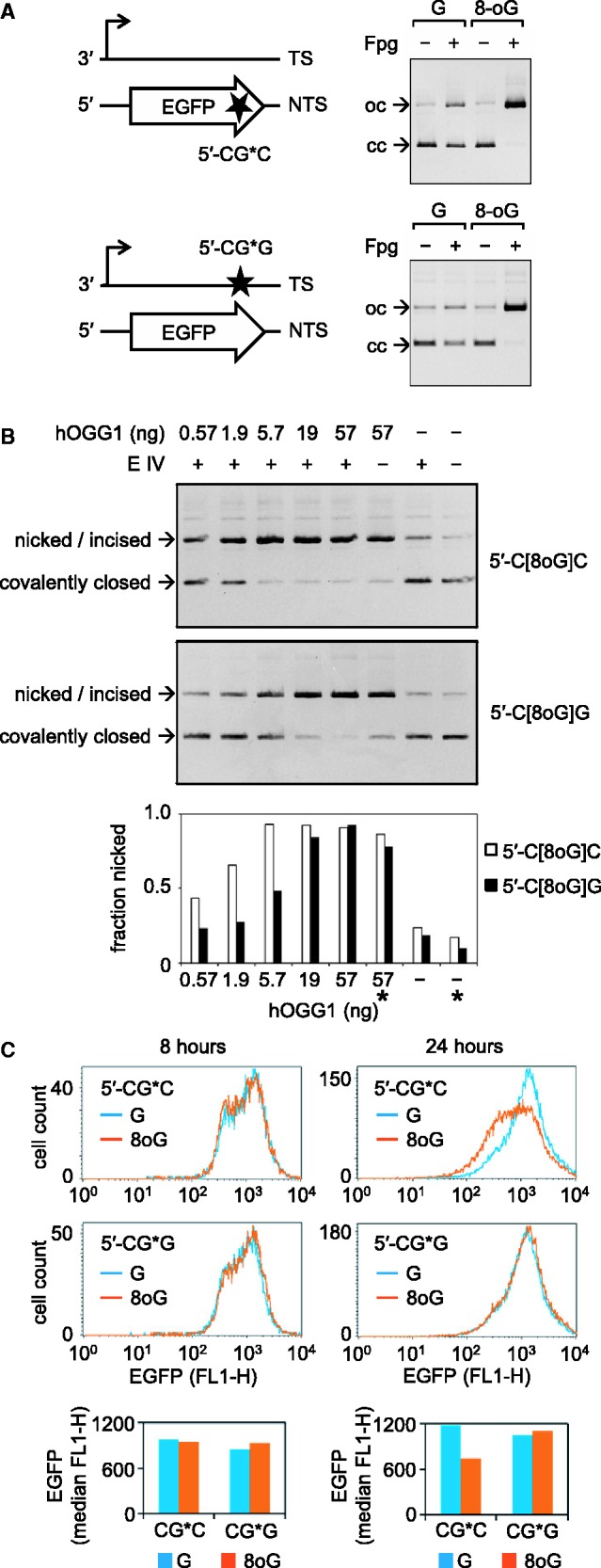
Analyses of vectors containing single 8-oxoG in the different sequence contexts and the opposing DNA strands. (**A**) Schematic representation of the positions (stars) and the immediate sequence contexts of 8-oxoG or G (G*) along with the analyses of vector DNA (pZAJ-3w-AGC) containing either G or 8-oxoG in the indicated positions. (**B**) Analyses of incision of the indicated plasmid substrates over the range of concentrations of human OGG1 protein in combination with endonuclease IV (E IV). Bars below the gel show quantification of DNA present in bands. Asterisks denote samples incubated without endonuclease IV. (**C**) Effects of single 8-oxoG in the indicated positions on the EGFP expression in HeLa cells. Fluorescence distribution plots and median EGFP fluorescence (below) for a typical experiment.

We previously reported that single 8-oxoG situated in the transcribed DNA strand within the EGFP-coding region of another vector has a much smaller capacity to inhibit both gene transcription and the EGFP protein expression in HeLa cells than an 8-oxoG closely located in the opposing DNA strand (10). Therefore, we were surprised as no difference between the DNA strands was found now for the lesions positioned in the 5′- and the 3′-UTR of the gene (Figure 1 and Supplementary Figure S2). The simplest explanation for this apparent contradiction would be that both the efficiency of excision of 8-oxoG and the resulting inhibition of transcription could depend on the DNA sequence surrounding the lesion—the parameter that has been deliberately kept constant in the present study (Figure 1), but not in the previous report (10). We therefore tested the *in vitro* cleavage activity of purified human OGG1 protein (OGG1) on covalently closed plasmid DNA substrates containing single 8-oxoG placed in two different sequence contexts available in the EGFP coding sequence (Figure 3A).

OGG1 is a bifunctional (DNA glycosylase/AP lyase) enzyme, which performs both hydrolytic removal of the damaged base and subsequent cleavage of the phosphodiester bond 3′ to the generated AP site (22); however, the DNA strand cleavage step has a much slower rate (33). We therefore performed the OGG1 cleavage reactions in the presence of an excess of endonuclease IV, to convert the AP sites underprocessed by OGG1 into strand breaks. By comparison of the cleavage rates over the range of OGG1 concentrations, we found that incision of the substrate containing single 8-oxoG within a 5′-C[8oxoG]C sequence is at least three times more efficient than of the substrate containing 8-oxoG in the 5′-C[8oxoG]G context in the complementary DNA strand (Figure 3B).

We further performed expression analyses of DNA containing single 8-oxoG in the two different sequence contexts by transfection of the corresponding plasmid vectors into HeLa cells. The results showed that both vectors were initially (8 h post-transfection) expressed at the same levels as the corresponding control vectors obtained by incorporation of the unmodified oligonucleotides. However, the expression of the vector containing 8-oxoG in the 5′-C[8oxoG]C context significantly declined by 24 h post-transfection, whereas the expression of the 5′-C[8oxoG]G-vector remained essentially unchanged in comparison with the vector containing the equivalent unmodified oligonucleotide (Figure 3C). Given that the inhibitory effect of single 8-oxoG on transcription is proportional to the excision efficiency, we infer that—also in cells—the excision of the 5′-C[8oxoG]C-substrate occurs with higher efficiency than of the 5′-C[8oxoG]G-substrate.

### Cellular effects of 8-oxoG depend on small number of adjacent nucleotides but not on the long-range DNA sequence or orientation with respect to the direction of transcription

**Figure 4.**
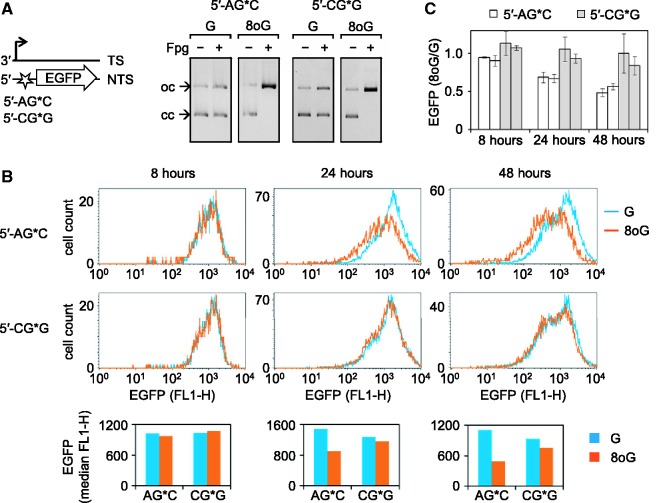
Effect of the DNA sequence around single 8-oxoG on the gene expression. (**A**) Vectors containing either G or 8-oxoG (G*) in the same position (star) but in different sequence contexts, as indicated. (**B** and **C**) Effects of single 8-oxoG in the specified sequence contexts on the EGFP expression in HeLa cells. (B) Fluorescence distribution plots and median EGFP fluorescence (below) for a typical experiment. (C) Relative EGFP expression summarized for two independently prepared 8-oxoG/G substrate pairs (individual bars, each representing mean of three transfection experiments +/− SD). See also Supplementary Figure S4.

To investigate whether the magnitude of the inhibitory effect of 8-oxoG on transcription is influenced only by nearby nucleotides or by a longer range DNA sequence, we further modified the vector sequences by artificially placing the 12-nt 5′-CAGGGCG*GACTG motif (where 8-oxoG was refractory to the excision by OGG1) into the 5′-UTR of the EGFP gene, i.e. >600 bp upstream from the original position in the gene. In addition, we inverted the strand orientation of the 12mer, now placing it into the non-transcribed DNA strand (Supplementary Figure S1). We then compared expression of the newly constructed vectors containing G or 8-oxoG (G*) in the 5′-CAGGGCG*GACTG sequence with the expression of already available constructs (Figure 1) containing G or 8-oxoG in the 5′-TTCGCTAG*CACG context and the otherwise identical DNA sequence. Independently of the DNA sequence, the 8-oxoG-containing vectors and the corresponding control constructs obtained by incorporation of the respective unmodified oligonucleotides showed identical expression levels in HeLa cells at early time (8 h) after transfections (Figure 4). Subsequent incubation resulted in a progressive decline of expression of the 5′-A[8-oxoG]C construct, which had a much stronger magnitude than the decrease observed in the case of the 5′-C[8-oxoG]G vector. We once more modified the vector sequence to compare the same two sequence contexts of 8-oxoG, placed this time in the opposing DNA strand (the transcribed strand of the gene)—with the same result (Supplementary Figure S4). We therefore conclude that different cellular processing and biological effects of differently located 8-oxoG are defined by DNA sequence confined to ≤11 nt surrounding the lesion (loosely a single turn of the DNA helix). Once again, orientation of the affected DNA strand with respect to the direction of transcription is unimportant.

### Excision rates of 8-oxoG are defined by the neighbouring nucleobases within a single helical turn of DNA

**Figure 5.**
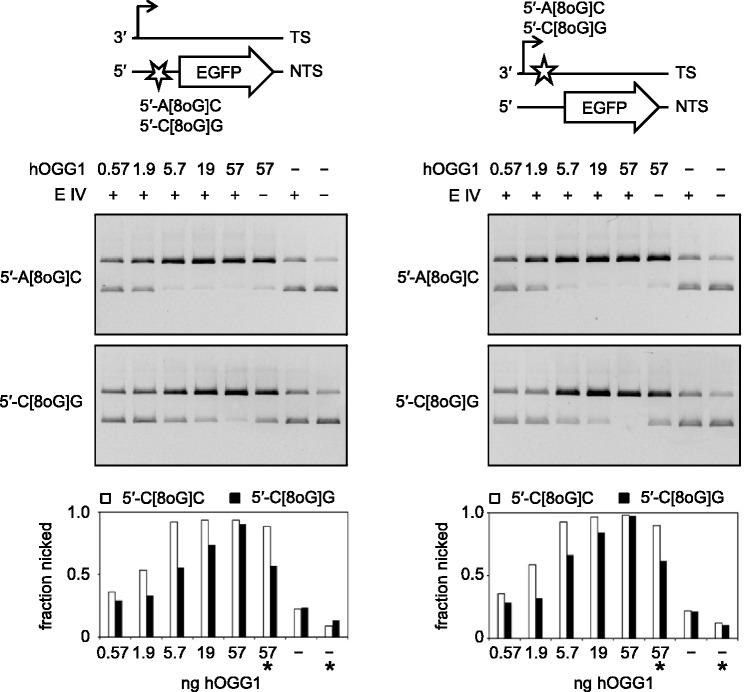
Incision of plasmid substrates containing single 8-oxoG in the specified sequence contexts by purified human OGG1 protein. Synthetic oligonucleotides containing G or 8-oxoG were inserted into different DNA strands, as indicated. Fractions of incised DNA were determined by molecular imaging analyses from the relative amounts of the covalently closed (lower band) and the open circular (upper band) forms of plasmid DNA. Endonuclease IV (E IV) was added to all samples except of those indicated by asterisks.

Given that the inhibited transcription of vector DNA containing single 8-oxoG fully depends on the damage excision by OGG1 (Figure 2), the different degrees of inhibition of transcription between the 5′-A[8-oxoG]C and 5′-C[8-oxoG]G sequences (Figure 4) infer that the excision efficiencies also must be different. These were tested in a direct assay with purified human OGG1 protein and the plasmid substrates, which contained single 8-oxoG in the two sequence contexts (5′-CAGGGC[8-oxoG]GACTG or 5′-TTCGCTA[8-oxoG]CACG) in either of the DNA strands. The results show that the incision efficiencies are at least 3-fold higher for each of the two 5′-A[8-oxoG]C-substrates than for the corresponding 5′-C[8-oxoG]G-substrates, as judged by the amounts of OGG1 required to induce the same degree of conversion of covalently closed circular plasmid DNA into the nicked form (Figure 5). On the other hand, the incision rates of the same DNA sequences in different strands of vector DNA are similar. In the absence of endonuclease IV, human OGG1 exhibits a slightly decreased strand cleavage activity, in agreement with the catalytic kinetics reported previously (33). Nonetheless, the incision rates of both 5′-A[8-oxoG]C substrates remain substantially higher than of the 5′-C[8-oxoG]G-substrates (Figure 5, lanes labelled with the asterisks). Thus, human OGG1 protein exhibits a pronounced selectivity of excision, which is largely defined by ≤11 nt surrounding the substrate 8-oxoG.

**Figure 6.**
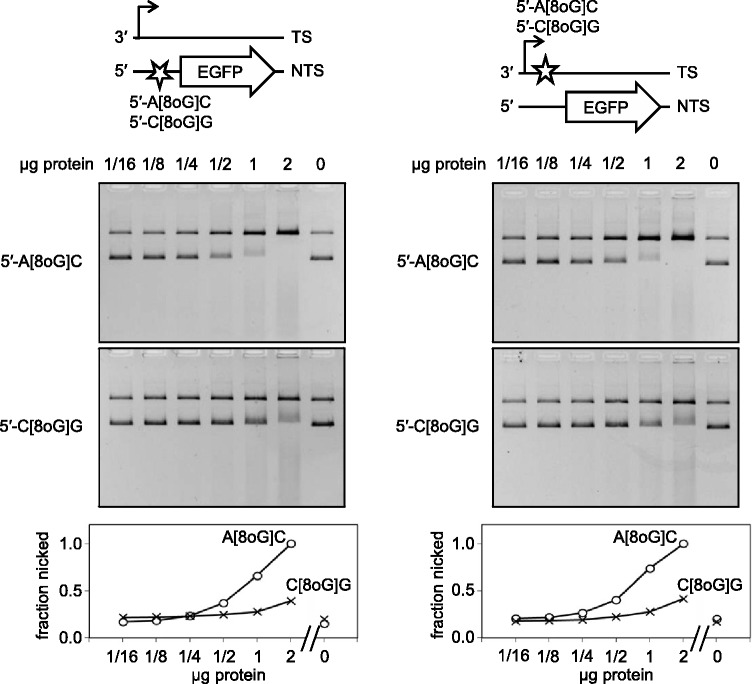
Incision of plasmid substrates containing single synthetic 8-oxoG in the specified sequence contexts by protein extract of HeLa cells overexpressing OGG1. Synthetic oligonucleotides containing G or 8-oxoG were inserted into different DNA strands, as indicated. Fractions of incised DNA were determined by molecular imaging analyses. Low molecular weight nucleic acid in gels originates from the cell extract. See also Supplementary Figure S5.

It has been reported that catalytic activity of OGG1 is enhanced or modulated by other proteins, most notably by the AP endonuclease APE1, which obsoletes the AP lyase step, thus increasing the OGG1 turnover (34,35). To allow such protein interactions, we further used cell extracts instead of the purified OGG1 to measure the excision rates of 8-oxoG from different plasmid substrates. Because of the excessive band shifting caused by DNA-binding proteins in the extracts obtained from HeLa cells, the naturally present incision activity was barely detectable. We observed weak incision of both 5′-A[8-oxoG]C substrates but not of the 5′-C[8-oxoG]G-substrates (Supplementary Figure S5). For a more quantitative comparison, we next used protein extracts from cells overexpressing human OGG1, which contained sufficient excision and incision activities (Figure 6). The incision of both substrates containing single 8-oxoG in the 5′-A[8-oxoG]C context was ∼4 times more efficient than the incision of the 5′-C[8-oxoG]G-substrates. This finding is coherent with the excision preferences of the purified OGG1 protein (Figure 5). Altogether, the results show that the excision step of BER of 8-oxoG is generally disfavoured within the 5′-CAGGGC[8-oxoG]GACTG motif and thus explain the influence of the surrounding DNA sequence on the degree of inhibition of the EGFP gene transcription by single 8-oxoG, which was documented in preceding experiments (Figures 3 and 4).

### DNA sequence is responsible for the excision bias between two 8-oxoG bases simultaneously situated in the opposite strands of the same DNA molecule

**Figure 7.**
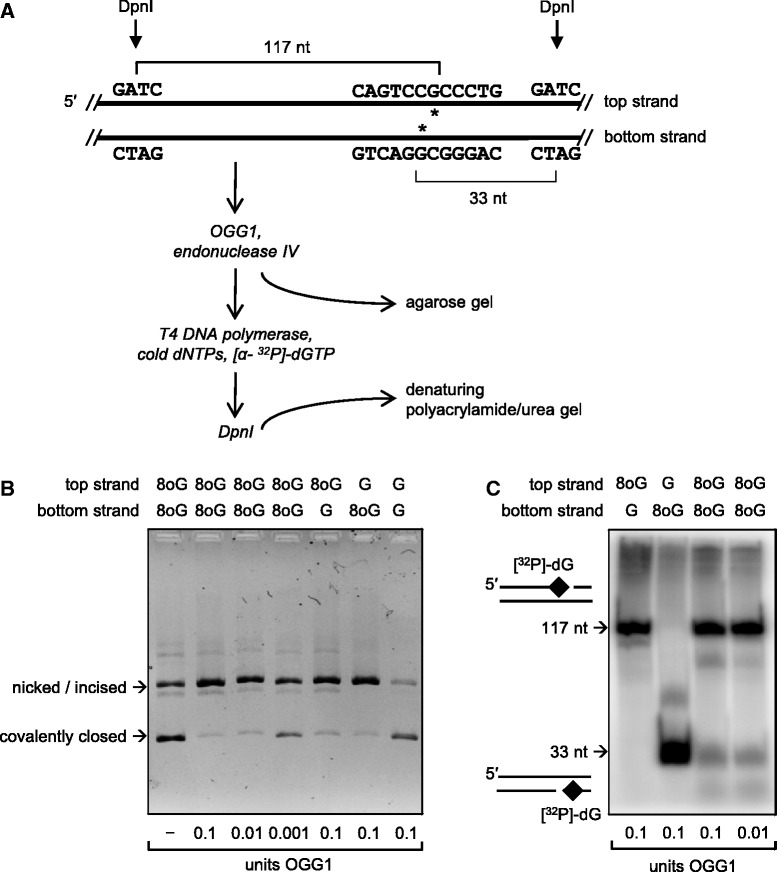
Determination of the hierarchy of excision of a clustered DNA lesion consisting of two 8-oxoG bases in the opposing DNA strands. (**A**) Map of a fragment of the plasmid substrate containing two 8-oxoG bases (asterisks) within the indicated sequence elements in the opposing DNA strands. (**B**) Agarose gel demonstrating the incision of plasmid substrates containing various combinations of G and 8-oxoG in top and bottom DNA strands by the indicated amounts of OGG1. (**C**) DNA strand mapping of the excised 8-oxoG by 3′-labelling with [α- ^32^P]-dGTP. See also Supplementary Figure S6.

Recognition of 8-oxoG by OGG1 requires bending of the DNA molecule within an ‘interrogation complex’, which results in flipping of the base out of the helical stack (36). For an 8-oxoG/C base pair in B-DNA, the efficiency of such structural transition can be influenced by the strength of hydrophobic stacking interactions with bases directly adjacent to 8-oxoG and perhaps by the somewhat broader local base composition, inasmuch as it defines the thermodynamic stability and rigidity of the DNA helix (37,38). However, certain DNA sequences adopt conformations different from the canonical B-form DNA, which can interfere with damage recognition by repair enzymes including OGG1 (39–41). To investigate, whether the inefficient base excision in the 5′-CAGGGC[8-oxoG]GACTG sequence is caused by a non-canonical DNA structure, we constructed a plasmid substrate containing a clustered lesion composed of two 8-oxoG bases located within a single CpG dinucleotide in the opposing DNA strands (Figure 7A, Supplementary Figure S6). We reasoned that if the inefficient excision observed at the 5′-CAGGGC[8-oxoG]GACTG motif was imposed by a peculiar conformation adopted by this DNA sequence, then the excision of an 8-oxoG in the opposing DNA strand must be equally inefficient.

We found that the bacterial Fpg enzyme efficiently incises one DNA strand of the substrate containing the Cp[8-oxoG]/Cp[8-oxoG] clustered lesion, whereas the second incision is strongly inhibited (Supplementary Figure S6). Similarly, only one DNA strand can be incised by human OGG1 even in the presence of a manifold excess of the enzyme (supplemented with endonuclease IV), as concluded from detection of the nicked form of plasmid DNA in the agarose gel but not of the linear form, which would derive from incision of both DNA strands (Figure 7B). This result is in full agreement with the previous report that excision of 8-oxoG by human OGG1 is inhibited by a strand break in the opposing DNA strand (42). We further used this property of the OGG1 cleavage to compare the excision rates between the DNA strands each containing 8-oxoG in a distinctive (non-palindromic) sequence context. The incision products of the plasmid substrates containing single or clustered 8-oxoG were labelled with [α-^32^P]-dGTP and digested with DpnI (as outlined in Figure 7A) to enable strand-specific mapping of the excised 8-oxoG. It turned out that 8-oxoG excision within the Cp[8-oxoG]/Cp[8-oxoG] bistranded cluster is remarkably strand-specific: the 5′-CAGTCC[8-oxoG]CCCTG sequence in one DNA strand was incised 9.2 times more often than its complementary 5′-CAGGGC[8-oxoG]GACTG sequence in another strand. Thus, for lesions located within the same molecule, the excision efficiencies are solely defined by the nucleotide sequence and not by average local base content or by the geometry or overall thermodynamic stability of the DNA helix.

EGFP expression analyses in HeLa cells showed that the Cp[8-oxoG]/Cp[8-oxoG] bistranded cluster lesion has a more pronounced negative effect on the gene expression than a single 8-oxoG in the vector DNA (Supplementary Figure S6). We suggest that recognition and excision of the clustered lesion by OGG1 might be accelerated in comparison with substrates containing single 8-oxoG. In line with this interpretation, a more rapid decrease of the gene expression (already observable after 8 h) occurred in the case of the clustered 8-oxoG.

## DISCUSSION

As an enzyme catalysing DNA depurination and strand cleavage, OGG1 needs an extraordinary substrate specificity to avoid self-induced genotoxicity. Studies with oligonucleotide substrates revealed that excision of 8-oxoG by human OGG1 is only efficient when the lesion is present in double-stranded DNA. The enzyme has evolved a strong preference for a complementary cytosine in the opposing DNA strand, which is beneficial because the excision inhibition by unmatched bases prevents mutation (18,19,21,43). Various kinds of DNA damage in proximity to 8-oxoG (a mismatch, an abasic site or a single-strand break) also strongly inhibit the excision by OGG1 (42,44), which is regarded as the mechanism for prevention of cytotoxic secondary damage that may arise from attempted repair of such complex lesions. The same could be the reason for the inefficient excision of 8-oxoG situated close to the ends of linear DNA (45,46) or in DNA sequences forming partly single-stranded structures such as hairpins, D-loops or bulges (41,47,48). It has not been clear until now whether, besides the mentioned particular DNA structures, any DNA sequence determinants exist that can influence the catalytic activity of OGG1. Measurements of the kinetic parameters in the oligonucleotide substrates have predicted that sequence of 10–13 nt around 8-oxoG has influence on the OGG1 binding and catalytic rates (48). However, because of the major influence of DNA duplex stability on the excision parameters in this system, the significance of these data is difficult to extrapolate to long DNA of highly stable helical structure, as it is found in the genome.

Here, we report that excision of single 8-oxoG from high-molecular weight DNA is significantly dependent on the nucleotide sequence around the lesion. We demonstrated this for purified OGG1 (alone and in combination with endonuclease IV) and for human cell extracts. Moreover, the excision in cells is also affected by the DNA sequence surrounding the lesion in apparently the same way, as judged from the expression of vector DNA harbouring single 8-oxoG in human host cells. By the experimental design, the sequence determinant for the inefficient excision must lie in one or more base pairs between the positions −6 and +5 with respect to 8-oxoG. Alignment of the DNA sequence context in which the excision of 8-oxoG is relatively inefficient (5′-CAGGGC[8-oxoG]GACTG) against the two available reference DNA sequences (5′-CAGTCC[8-oxoG]CCCTG and 5′-TCGCTA[8-oxoG]CACGC) confines the critical sequence elements to as little as three guanine bases in the positions +1, −2 and −3 with respect to the position of 8-oxoG (underlined in the first sequence). Based on the known catalytic mechanism of OGG1 (37), it seems reasonable to propose that the hydrophobic stacking interactions with the adjacent guanine could disfavour transition of 8-oxoG into the extrahelical conformation (flipping out), whereas the upstream GG dinucleotide could enhance this effect by sustaining the GC-rich sequence around the lesion and thus contributing to local stabilization of DNA double helix. However, the asymmetry of the excision at the bistranded Cp[8-oxoG]/Cp[8-oxoG] clustered lesion provides a clue that the importance of local GC-content and overall helical stability is not yet decisive. Our data (Figure 7) show that, within the same helix, the G-rich DNA strand is excised with a much lower efficiency than the complementary C-rich strand. In view of the fact that extrusion of 8-oxoG from the DNA helix is coupled with strong bending of the DNA molecule (36,43), it is intriguing to speculate that the sequence context might set the preferred bending direction, which would favour the extrusion of one 8-oxoG but disfavour it for another 8-oxoG on the opposite side of the helix.

Our finding that BER of 8-oxoG is dependent on the sequence context predicts that the sequences repaired in cells at slower rates will generally accumulate more damage than the genome’s average. In replicating cells, such unrepaired damage has a chance to translate into mutations. It has been previously hypothesized that inefficient repair of certain genomic regions and sequences could account for heterogenous distribution of 8-oxoG and of the resulting mutations observed in human cells (7,29). In support of this notion, a recent study using artificially reconstituted dinucleosomal substrates revealed that the rate of excision of 8-oxoG by OGG1 is modulated in a position-dependent manner by chromatin components (49). Now, we demonstrate that DNA sequence itself imposes significant constraints on the excision of 8-oxoG as well.

Besides the possible impact on maintenance of genomic stability, DNA-sequence selectivity of BER has straightforward implications for gene transcription—just another essential biological function, which is affected by 8-oxoG. Our data clearly show that there is interference between BER and transcription if both processes occur simultaneously in mammalian cells. The findings that the degree of inhibition of the gene expression is proportional to the OGG1 protein level (Figure 2), and the sequence-specific excision activity (Figures 3–6) strongly suggest that transcription is affected in the post-excision stage of repair, for instance in result of encounter of transcription machineries with a single-strand break generated by OGG1. Such a scenario looks plausible, as single-strand breaks cause potent inhibition of transcription in cell-free systems (11,50) and in cells (51). The interference of BER with transcription implies that there might well be a negative selection pressure against excessive expression of OGG1, thus explaining the relatively high steady-state levels of 8-oxoG in the genome and rationalizing the necessity for the additional MUTYH-dependent antimutagenic pathway.

## SUPPLEMENTARY DATA

Supplementary Data are available at NAR Online, including [52–57].

## FUNDING

Deutsche Forschungsgemeinschaft (DFG, German Research Foundation) [KH 263/1, KH 263/2 to A.K.]. Funding for open access charge: Deutsche Forschungsgemeinschaft.

*Conflict of interest statement*. None declared.

## Supplementary Material

Supplementary Data

## References

[1] Lindahl T (1993). Instability and decay of the primary structure of DNA. Nature.

[2] Neeley WL, Essigmann JM (2006). Mechanisms of formation, genotoxicity, and mutation of guanine oxidation products. Chem. Res. Toxicol..

[3] Burrows CJ, Muller JG (1998). Oxidative nucleobase modifications leading to strand scission. Chem. Rev..

[4] Shibutani S, Takeshita M, Grollman AP (1991). Insertion of specific bases during DNA synthesis past the oxidation-damaged base 8-oxodG. Nature.

[5] Hsu GW, Ober M, Carell T, Beese LS (2004). Error-prone replication of oxidatively damaged DNA by a high-fidelity DNA polymerase. Nature.

[6] Maga G, Villani G, Crespan E, Wimmer U, Ferrari E, Bertocci B, Hubscher U (2007). 8-oxo-guanine bypass by human DNA polymerases in the presence of auxiliary proteins. Nature.

[7] Al-Tassan N, Chmiel NH, Maynard J, Fleming N, Livingston AL, Williams GT, Hodges AK, Davies DR, David SS, Sampson JR (2002). Inherited variants of MYH associated with somatic G:C—>T:A mutations in colorectal tumors. Nat. Genet..

[8] Markkanen E, Dorn J, Hubscher U (2013). MUTYH DNA glycosylase: the rationale for removing undamaged bases from the DNA. Front. Genet..

[9] Saxowsky TT, Meadows KL, Klungland A, Doetsch PW (2008). 8-Oxoguanine-mediated transcriptional mutagenesis causes Ras activation in mammalian cells. Proc. Natl Acad. Sci. USA.

[10] Kitsera N, Stathis D, Luhnsdorf B, Muller H, Carell T, Epe B, Khobta A (2011). 8-Oxo-7,8-dihydroguanine in DNA does not constitute a barrier to transcription, but is converted into transcription-blocking damage by OGG1. Nucleic Acids Res..

[11] Kathe SD, Shen GP, Wallace SS (2004). Single-stranded breaks in DNA but not oxidative DNA base damages block transcriptional elongation by RNA polymerase II in HeLa cell nuclear extracts. J. Biol. Chem..

[12] Larsen E, Kwon K, Coin F, Egly JM, Klungland A (2004). Transcription activities at 8-oxoG lesions in DNA. DNA Repair.

[13] Tornaletti S, Maeda LS, Kolodner RD, Hanawalt PC (2004). Effect of 8-oxoguanine on transcription elongation by T7 RNA polymerase and mammalian RNA polymerase II. DNA Repair.

[14] Charlet-Berguerand N, Feuerhahn S, Kong SE, Ziserman H, Conaway JW, Conaway R, Egly JM (2006). RNA polymerase II bypass of oxidative DNA damage is regulated by transcription elongation factors. EMBO J..

[15] Kuraoka I, Suzuki K, Ito S, Hayashida M, Kwei JS, Ikegami T, Handa H, Nakabeppu Y, Tanaka K (2007). RNA polymerase II bypasses 8-oxoguanine in the presence of transcription elongation factor TFIIS. DNA Repair.

[16] Aburatani H, Hippo Y, Ishida T, Takashima R, Matsuba C, Kodama T, Takao M, Yasui A, Yamamoto K, Asano M (1997). Cloning and characterization of mammalian 8-hydroxyguanine-specific DNA glycosylase/apurinic, apyrimidinic lyase, a functional mutM homologue. Cancer Res..

[17] Rosenquist TA, Zharkov DO, Grollman AP (1997). Cloning and characterization of a mammalian 8-oxoguanine DNA glycosylase. Proc. Natl Acad. Sci. USA.

[18] Lu R, Nash HM, Verdine GL (1997). A mammalian DNA repair enzyme that excises oxidatively damaged guanines maps to a locus frequently lost in lung cancer. Curr. Biol..

[19] Radicella JP, Dherin C, Desmaze C, Fox MS, Boiteux S (1997). Cloning and characterization of hOGG1, a human homolog of the OGG1 gene of *Saccharomyces cerevisiae*. Proc. Natl Acad. Sci. USA.

[20] Roldan-Arjona T, Wei YF, Carter KC, Klungland A, Anselmino C, Wang RP, Augustus M, Lindahl T (1997). Molecular cloning and functional expression of a human cDNA encoding the antimutator enzyme 8-hydroxyguanine-DNA glycosylase. Proc. Natl Acad. Sci. USA.

[21] Bjoras M, Luna L, Johnsen B, Hoff E, Haug T, Rognes T, Seeberg E (1997). Opposite base-dependent reactions of a human base excision repair enzyme on DNA containing 7,8-dihydro-8-oxoguanine and abasic sites. EMBO J..

[22] Nash HM, Lu R, Lane WS, Verdine GL (1997). The critical active-site amine of the human 8-oxoguanine DNA glycosylase, hOgg1: direct identification, ablation and chemical reconstitution. Chem. Biol..

[23] Fromme JC, Bruner SD, Yang W, Karplus M, Verdine GL (2003). Product-assisted catalysis in base-excision DNA repair. Nat. Struct. Biol..

[24] Klungland A, Rosewell I, Hollenbach S, Larsen E, Daly G, Epe B, Seeberg E, Lindahl T, Barnes DE (1999). Accumulation of premutagenic DNA lesions in mice defective in removal of oxidative base damage. Proc. Natl Acad. Sci. USA.

[25] Osterod M, Larsen E, Le Page F, Hengstler JG, Van Der Horst GT, Boiteux S, Klungland A, Epe B (2002). A global DNA repair mechanism involving the Cockayne syndrome B (CSB) gene product can prevent the *in vivo* accumulation of endogenous oxidative DNA base damage. Oncogene.

[26] Arai T, Kelly VP, Minowa O, Noda T, Nishimura S (2002). High accumulation of oxidative DNA damage, 8-hydroxyguanine, in Mmh/Ogg1 deficient mice by chronic oxidative stress. Carcinogenesis.

[27] Larsen E, Reite K, Nesse G, Gran C, Seeberg E, Klungland A (2006). Repair and mutagenesis at oxidized DNA lesions in the developing brain of wild-type and Ogg1-/- mice. Oncogene.

[28] Gedik CM, Collins A, Escodd (2005). Establishing the background level of base oxidation in human lymphocyte DNA: results of an interlaboratory validation study. FASEB J..

[29] Ohno M, Miura T, Furuichi M, Tominaga Y, Tsuchimoto D, Sakumi K, Nakabeppu Y (2006). A genome-wide distribution of 8-oxoguanine correlates with the preferred regions for recombination and single nucleotide polymorphism in the human genome. Genome Res..

[30] Amouroux R, Campalans A, Epe B, Radicella JP (2010). Oxidative stress triggers the preferential assembly of base excision repair complexes on open chromatin regions. Nucleic Acids Res..

[31] Lühnsdorf B, Kitsera N, Warken D, Lingg T, Epe B, Khobta A (2012). Generation of reporter plasmids containing defined base modifications in the DNA strand of choice. Anal. Biochem..

[32] Khobta A, Anderhub S, Kitsera N, Epe B (2010). Gene silencing induced by oxidative DNA base damage: association with local decrease of histone H4 acetylation in the promoter region. Nucleic Acids Res..

[33] Kuznetsov NA, Koval VV, Zharkov DO, Nevinsky GA, Douglas KT, Fedorova OS (2005). Kinetics of substrate recognition and cleavage by human 8-oxoguanine-DNA glycosylase. Nucleic Acids Res..

[34] Hill JW, Hazra TK, Izumi T, Mitra S (2001). Stimulation of human 8-oxoguanine-DNA glycosylase by AP-endonuclease: potential coordination of the initial steps in base excision repair. Nucleic Acids Res..

[35] Vidal AE, Hickson ID, Boiteux S, Radicella JP (2001). Mechanism of stimulation of the DNA glycosylase activity of hOGG1 by the major human AP endonuclease: bypass of the AP lyase activity step. Nucleic Acids Res..

[36] Chen L, Haushalter KA, Lieber CM, Verdine GL (2002). Direct visualization of a DNA glycosylase searching for damage. Chem. Biol..

[37] Friedman JI, Stivers JT (2010). Detection of damaged DNA bases by DNA glycosylase enzymes. Biochemistry.

[38] Singh SK, Szulik MW, Ganguly M, Khutsishvili I, Stone MP, Marky LA, Gold B (2011). Characterization of DNA with an 8-oxoguanine modification. Nucleic Acids Res..

[39] Zhao J, Bacolla A, Wang G, Vasquez KM (2010). Non-B DNA structure-induced genetic instability and evolution. Cell. Mol. Life Sci..

[40] Jarem DA, Wilson NR, Schermerhorn KM, Delaney S (2011). Incidence and persistence of 8-oxo-7,8-dihydroguanine within a hairpin intermediate exacerbates a toxic oxidation cycle associated with trinucleotide repeat expansion. DNA Repair.

[41] Rhee DB, Ghosh A, Lu J, Bohr VA, Liu Y (2011). Factors that influence telomeric oxidative base damage and repair by DNA glycosylase OGG1. DNA Repair.

[42] David-Cordonnier MH, Boiteux S, O'Neill P (2001). Efficiency of excision of 8-oxo-guanine within DNA clustered damage by XRS5 nuclear extracts and purified human OGG1 protein. Biochemistry.

[43] Bruner SD, Norman DP, Verdine GL (2000). Structural basis for recognition and repair of the endogenous mutagen 8-oxoguanine in DNA. Nature.

[44] Sassa A, Beard WA, Prasad R, Wilson SH (2012). DNA sequence context effects on the glycosylase activity of human 8-oxoguanine DNA glycosylase. J. Biol. Chem..

[45] Hirano T, Hirano H, Yamaguchi R, Asami S, Tsurudome Y, Kasai H (2001). Sequence specificity of the 8-hydroxyguanine repair activity in rat organs. J. Radiat. Res..

[46] Derevyanko AG, Endutkin AV, Ishchenko AA, Saparbaev MK, Zharkov DO (2012). Initiation of 8-oxoguanine base excision repair within trinucleotide tandem repeats. Biochemistry.

[47] Jarem DA, Wilson NR, Delaney S (2009). Structure-dependent DNA damage and repair in a trinucleotide repeat sequence. Biochemistry.

[48] Kirpota OO, Endutkin AV, Ponomarenko MP, Ponomarenko PM, Zharkov DO, Nevinsky GA (2011). Thermodynamic and kinetic basis for recognition and repair of 8-oxoguanine in DNA by human 8-oxoguanine-DNA glycosylase. Nucleic Acids Res..

[49] Menoni H, Shukla MS, Gerson V, Dimitrov S, Angelov D (2012). Base excision repair of 8-oxoG in dinucleosomes. Nucleic Acids Res..

[50] Roy D, Zhang Z, Lu Z, Hsieh CL, Lieber MR (2010). Competition between the RNA transcript and the nontemplate DNA strand during R-loop formation *in vitro*: a nick can serve as a strong R-loop initiation site. Mol. Cell. Biol..

[51] Khobta A, Lingg T, Schulz I, Warken D, Kitsera N, Epe B (2010). Mouse CSB protein is important for gene expression in the presence of a single-strand break in the non-transcribed DNA strand. DNA Repair.

[52] Khobta A, Kitsera N, Speckmann B, Epe B (2009). 8-Oxoguanine DNA glycosylase (Ogg1) causes a transcriptional inactivation of damaged DNA in the absence of functional Cockayne syndrome B (Csb) protein. DNA Repair.

[53] Shareef MM, Dancea HC, Gross JL, Myers TT, Griggs WW, Ahmed MM, Sheldon DG (2008). A noncommercial polymerase chain reaction-based method to approach one hundred percent recombinant clone selection efficiency. Anal. Biochem..

[54] Kohn KW, Erickson LC, Ewig RA, Friedman CA (1976). Fractionation of DNA from mammalian cells by alkaline elution. Biochemistry.

[55] Epe B, Pflaum M, Boiteux S (1993). DNA damage induced by photosensitizers in ellular and cell-free systems. Mutat. Res..

[56] Epe B, Hegler J (1994). Oxidative DNA damage: endonuclease fingerprinting. Methods Enzymol..

[57] Boiteux S, O'Connor TR, Lederer F, Gouyette A, Laval J (1990). Homogeneous *Escherichia coli* FPG protein A DNA glycosylase which excises imidazole ring-opened purines and nicks DNA at apurinic/apyrimidinic sites. J. Biol. Chem..

