# Supplementary material for: Modulation of base excision repair of 8-oxoguanine by the nucleotide sequence
Source: Nucleic Acids Res. 2013 Jul 17;41(18):8559–71. doi: 10.1093/nar/gkt620 (PMC3794583; doi:10.1093/nar/gkt620)
Supplement: Supplementary Data [file supp_41_18_8559__index.html]

Modulation of base excision repair of 8-oxoguanine by the nucleotide sequence — Modulation of base excision repair of 8-oxoguanine by the nucleotide sequence — Supplementary Data 

# Modulation of base excision repair of 8-oxoguanine by the nucleotide sequence

## 

files

**Files in this Data Supplement:**

- Supplementary Data - pdf file
