# Supplementary material for: Modulation of base excision repair of 8-oxoguanine by the nucleotide sequence
Source: Nucleic Acids Res. 2013 Jul 17;41(18):8559–71. doi: 10.1093/nar/gkt620 (PMC3794583; doi:10.1093/nar/gkt620)
Supplement: Supplementary Data [file supp_gkt620_nar-01443-d-2013-File009.pdf]

Modulation of base excision repair of 8-oxoguanine by the nucleotide sequence

Julia Allgayer, Nataliya Kitsera, Carina von der Lippen, Bernd Epe, and Andriy Khobta

SUPPLEMENTARY DATA

```
pZAJ-5w-AGC    CTAGACATTGCTTCGCTAGCACGCATTGCCTA
pZAJ-5c-AGC    CTAGAGCAATGCGTGCTAGCGAAGCAATGCTA
pZAJ-5w-CGG    CTAGACATTGCAGGGCGGACTGACATTGCCTA
pZAJ-5c-CGG    CTAGAGCAATGTCAGTCCGCCCTGCAATGCTA

                                                                        →EGFP coding sequence
GTCTATATAAGCAGAGCTGGTTTAGTGAACCGTCAGATCCGctagcgctaCCGGTCGCCACCATGGTGAGCAAGGGCGAGGAGCTGTTCACCGGGGTGGT

GCCCATCCTGGTCGAGCTGGACGGCGACGTAAACGGCCACAAGTTCAGCGTGTCCGGCGAGGGCGAGGGCGATGCCACCTACGGCAAGCTGACCCTGAAG

TTCATCTGCACCACCGGCAAGCTGCCCGTGCCCTGGCCCACCCTCGTGACCACCCTGACCTACGGCGTGCAGTGCTTCAGCCGCTACCCCGACCACATGA

AGCAGCACGACTTCTTCAAGTCCGCCATGCCCGAAGGCTACGTCCAGGAGCGCACCATCTTCTTCAAGGACGACGGCAACTACAAGACCCGCGCCGAGGT

GAAGTTCGAGGGCGACACCCTGGTGAACCGCATCGAGCTGAAGGGCATCGACTTCAAGGAGGACGGCAACATCCTGGGGCACAAGCTGGAGTACAACTAC

AACAGCCACAACGTCTATATCATGGCCGACAAGCAGAAGAACGGCATCAAGGTGAACTTCAAGATCCGCCACAACATCGAGGACGGCAGCGTGCAGCTCG

CCGACCACTACCAGCAGAACACCCCCATCGGCGACGGCCCCGTGCTGCTGCCCGACAACCACTACCTGAGCACCCAGTCCGCCCTGAGCAAAGACCCCAA
                                                               EGFP coding sequence→|
CGAGAAGCGCGATCACATGGTCCTGCTGGAGTTCGTGACCGCCGCCGGGATCACTCTCGGCATGGACGAGCTGTACAAGTACTAGCATCTACACATTGAT

CCTAGCAGAAGCACAGGCTGCAGGGTGACGGTCCATCCCGCTCTCCTGGGCACAAGGCATGGGCAGCGTGCCATCATCCTGCTCCTCCACCTCCGGCGGG

AAGCCATGGCTCTGGATCTGCTTCATGAGTGAGTACTCAGATCTCGAGCTCAAGCTTCGAATTCTGCAGtcgacggtacCGCGGGCCCGGGATCCACCGG

                                      pZAJ-3w-AGC    TCGAGCATTGCTTCGCTAGCACGCATTGCGGTAC
                                      pZAJ-3c-AGC    TCGAGGCAATGCGTGCTAGCGAAGCAATGGGTAC
```

Figure S1. Modifications done to pZAJ vector in order to enable site-specific incorporation of single 8-oxoG in various positions and in different DNA sequence contexts. DNA sequence fragment of the pZAJ vector is shown. Transcribed region of the EGFP gene is underlined. The translation initiation and termination codons are shown in bold. DNA sequences suited for incorporation of synthetic oligonucleotides containing 8-oxoG and the names assigned to the newly-obtained vectors are shown in the insets above and below the sequence (for the 5′- and 3′-untranslated gene regions, respectively). Wavy underlines in the insets indicate the sequence recognised by BsrDI. Positions subsequently used for the incorporation of 8-oxoG are red-highlighted. The sequences concomitantly deleted from the pZAJ vector are shown by grey background.

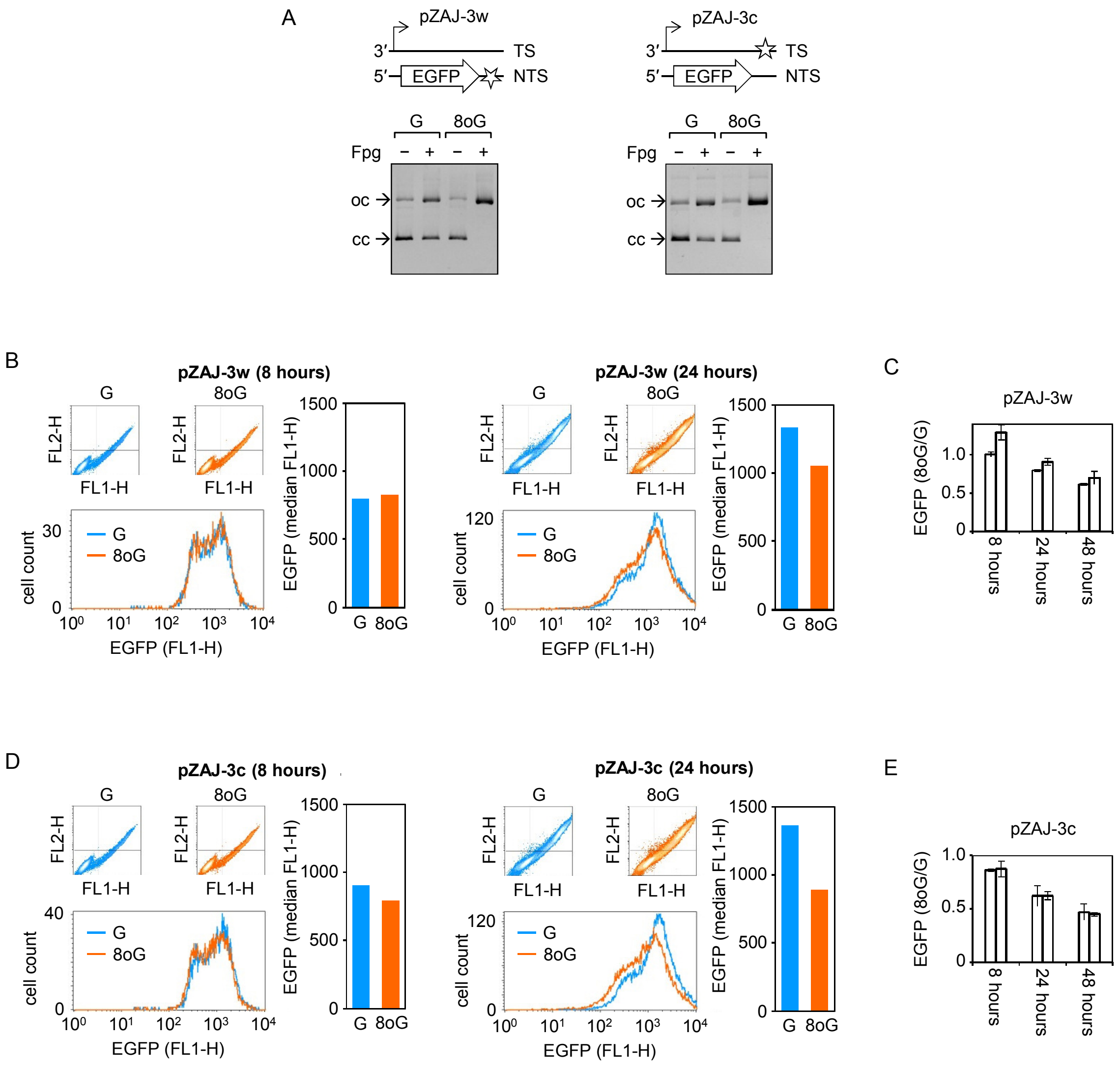

Figure S2. Effect of single 8-oxoG in the 3′-untranslated region (3′-UTR) on expression of the EGFP-coding gene in Hela cells. (A) Analyses of vector DNA containing either dG or 8-oxodG by incubation with Fpg DNA glycosylase. Diagrams illustrate position of 8-oxoG (star) with respect to transcription start (broken arrow) and the protein-coding DNA sequence (arrow). (B–E) Flow cytometric analyses of HeLa cells co-transfected with equal amounts of a vector encoding for DsRed-Monomer and the indicated EGFP encoding plasmids. (B and D) Results of typical experiments. (C and E) Relative EGFP expression summarised for two independently prepared 8-oxoG/G substrate pairs (separate bars, each representing mean of 3 transfection experiments +/-SD.)

A

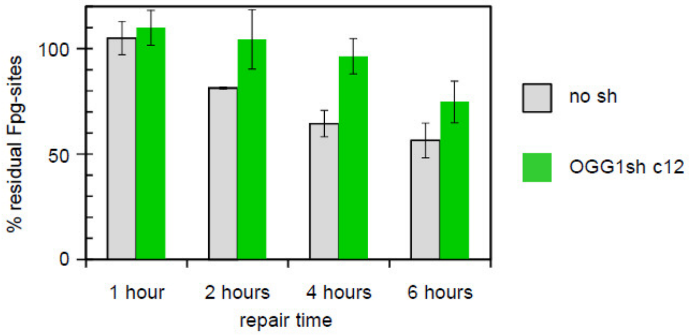

B

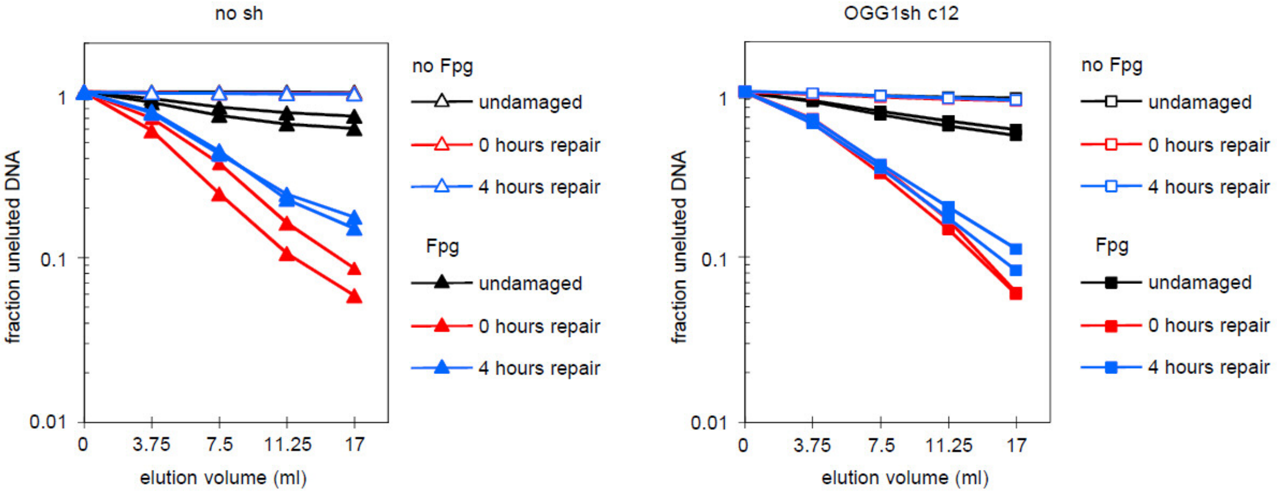

Figure S3. Retarded repair of oxidative damage generated by $KBrO_3$ in genomic DNA of HeLa-derived cell lines stably expressing OGG1 shRNA (OGG1sh c12) compared to the isogenic clone transfected with the empty vector (no sh). (A) Percentages of residual Fpg-sensitive sites in genome of damaged cells after the indicated repair times (mean of three independent experiments +/-SD). Damage present at the moment of withdrawal of potassium bromate (0 hours repair) corresponds to 100%. (B) Representative alkaline elution profiles of genome DNA incubated on filters with or without Fpg. Rates of elution are proportional to the amounts of SSB. All DNA remained on filters in samples incubated without Fpg because SSB are not induced by the low dose of $KBrO_3$ used here. Elution rates in samples treated with Fpg are proportional to the levels of genomic 8-oxoG (incised during the filter incubation).

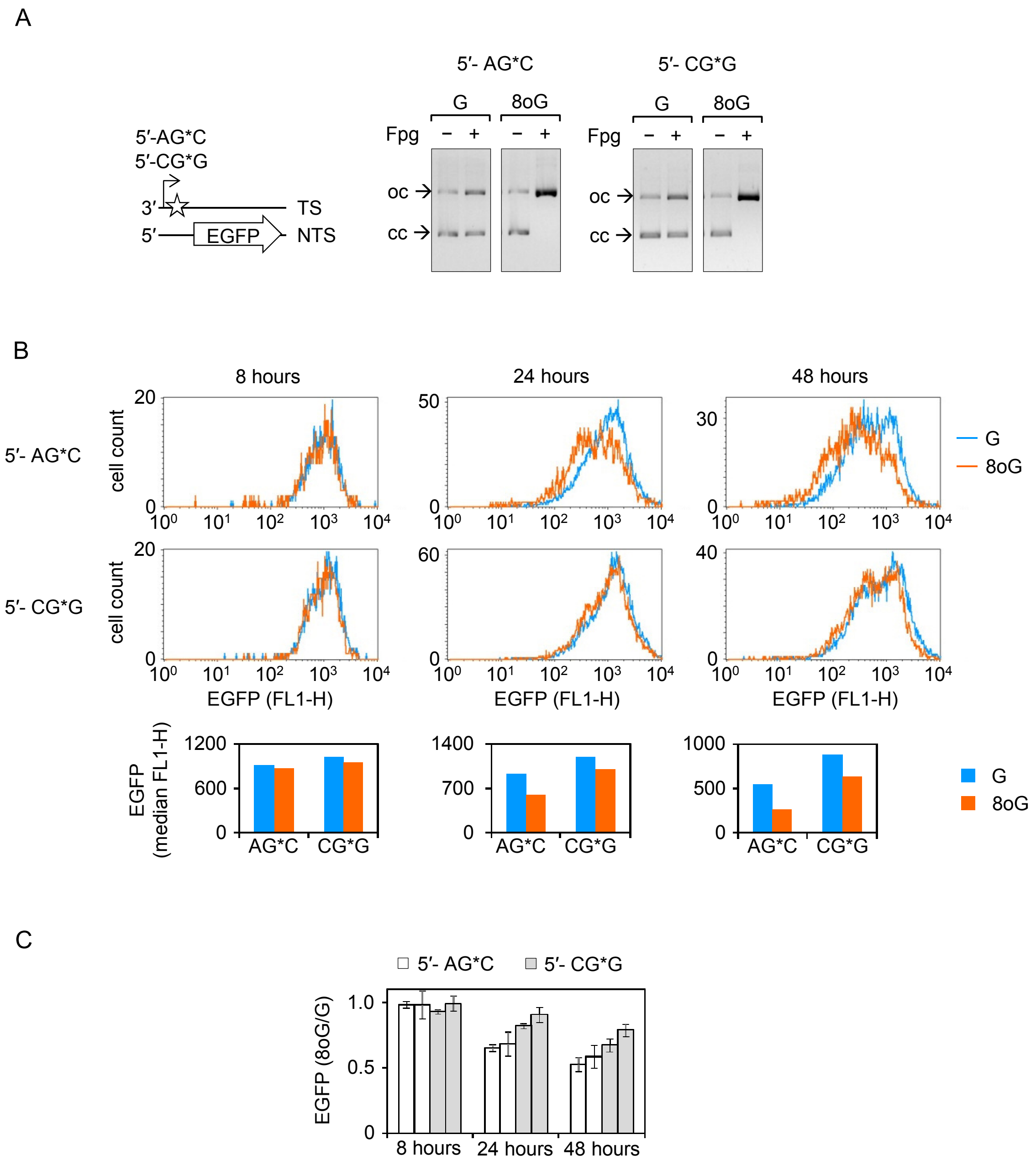

Figure S4. Effect of the DNA sequence around single 8-oxoG in the transcribed DNA strand (TS) on the EGFP gene expression. (A) Vectors containing either G or 8-oxoG (G*) in the same position (star) but in different sequence contexts. (B and C) Effects of single 8-oxoG in different sequence contexts on the EGFP expression in HeLa cells. (B) Fluorescence distribution plots and median EGFP fluorescence (below) for a typical experiment. (C) Relative EGFP expression summarised for two independently prepared 8-oxoG/G substrate pairs (individual bars, each representing mean of 3 transfection experiments +/-SD).

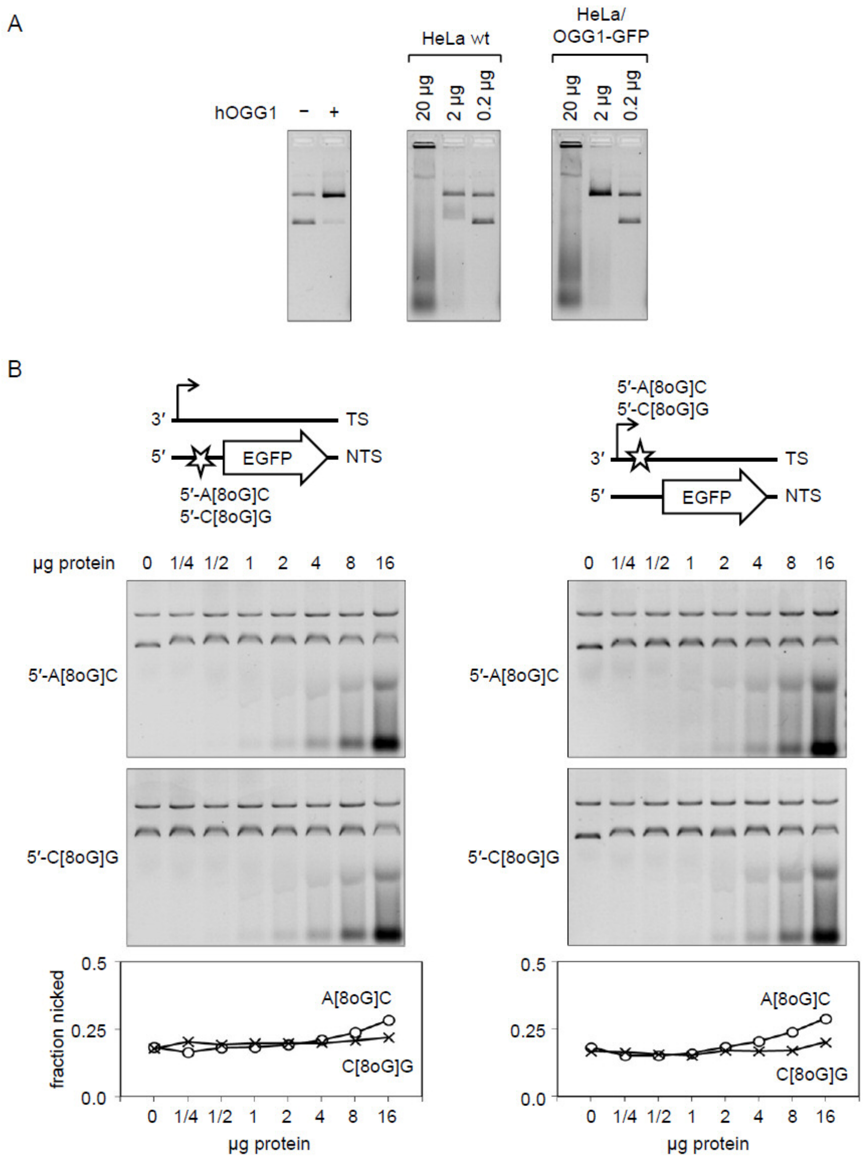

Figure S5. Incision of plasmid substrates containing single synthetic 8-oxoG in the specified positions and sequence contexts by protein extract of HeLa cells. (A) Incision generated by pure human OGG1 protein and by the indicated amounts of the protein extracts in the pZAJ-5w-AGC plasmid substrate containing single 8-oxoG. Cell extracts induce a strong band-shifting which interferes with the detection of incision. (B) Analyses of incision of the indicated plasmid substrates in gels containing 0.1% SDS. Cleavage was performed with the wild-type extracts (total protein amounts are indicated). Fractions of incised DNA were determined by molecular imaging analyses. Low molecular weight nucleic acid in gels originates from the cell extract.

A

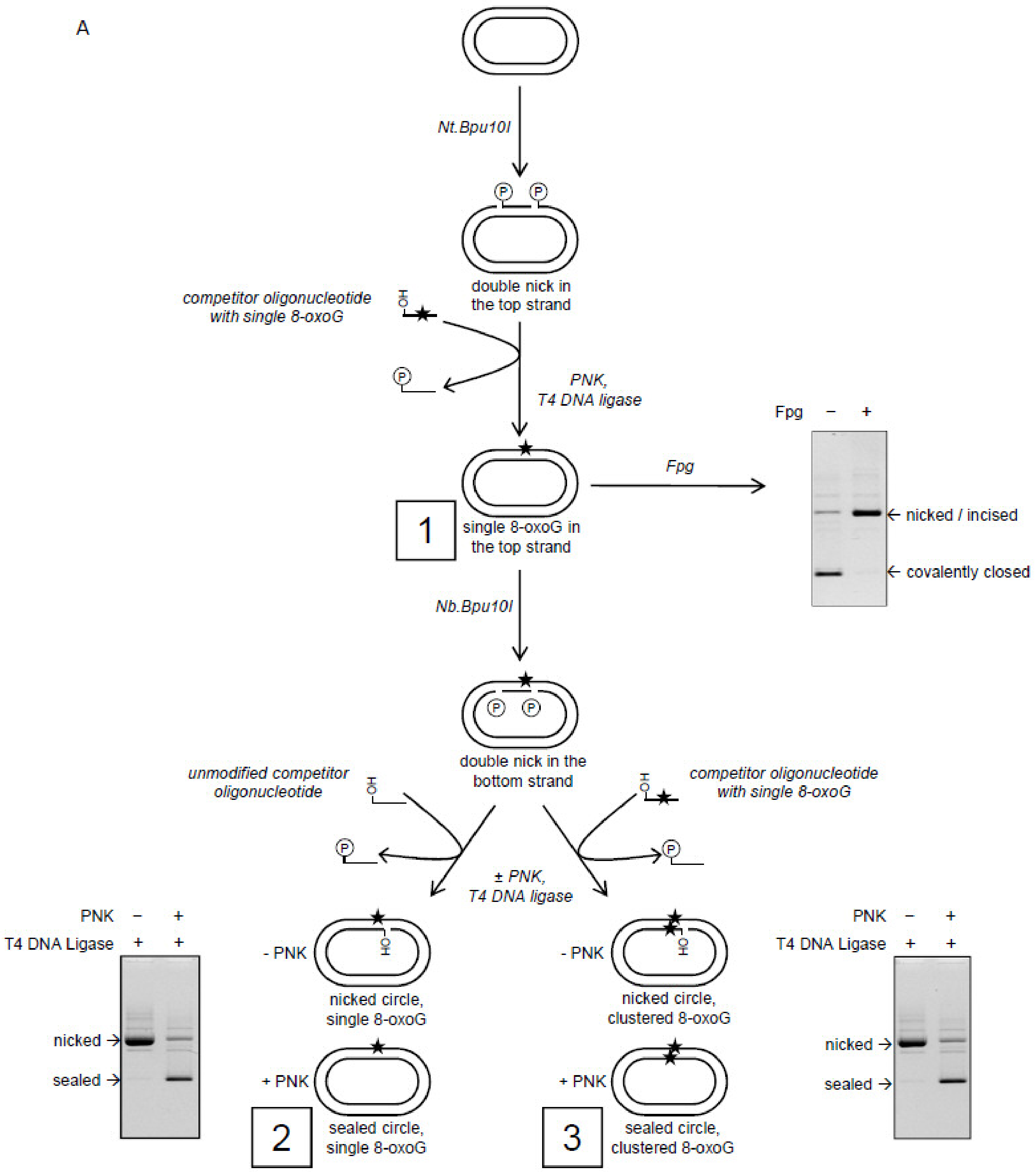

Nt.Bpu10I
double nick in the top strand
competitor oligonucleotide with single 8-oxoG
PNK, T4 DNA ligase
1
single 8-oxoG in the top strand
Fpg
Fpg – +
nicked / incised
covalently closed
Nb.Bpu10I
double nick in the bottom strand
unmodified competitor oligonucleotide
competitor oligonucleotide with single 8-oxoG
± PNK, T4 DNA ligase
PNK – +
T4 DNA Ligase + +
nicked
sealed
- PNK
nicked circle, single 8-oxoG
+ PNK
2
sealed circle, single 8-oxoG
- PNK
nicked circle, clustered 8-oxoG
+ PNK
3
sealed circle, clustered 8-oxoG
PNK – +
T4 DNA Ligase + +
nicked
sealed

B

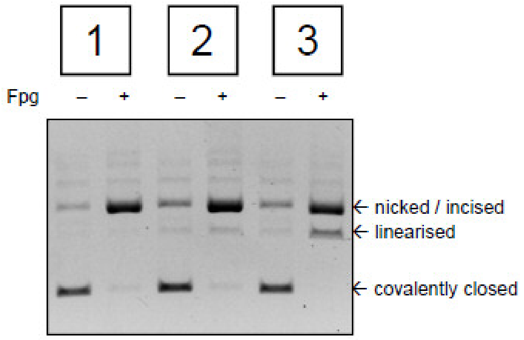

1
2
3
Fpg – + – + – +
nicked / incised
linearised
covalently closed

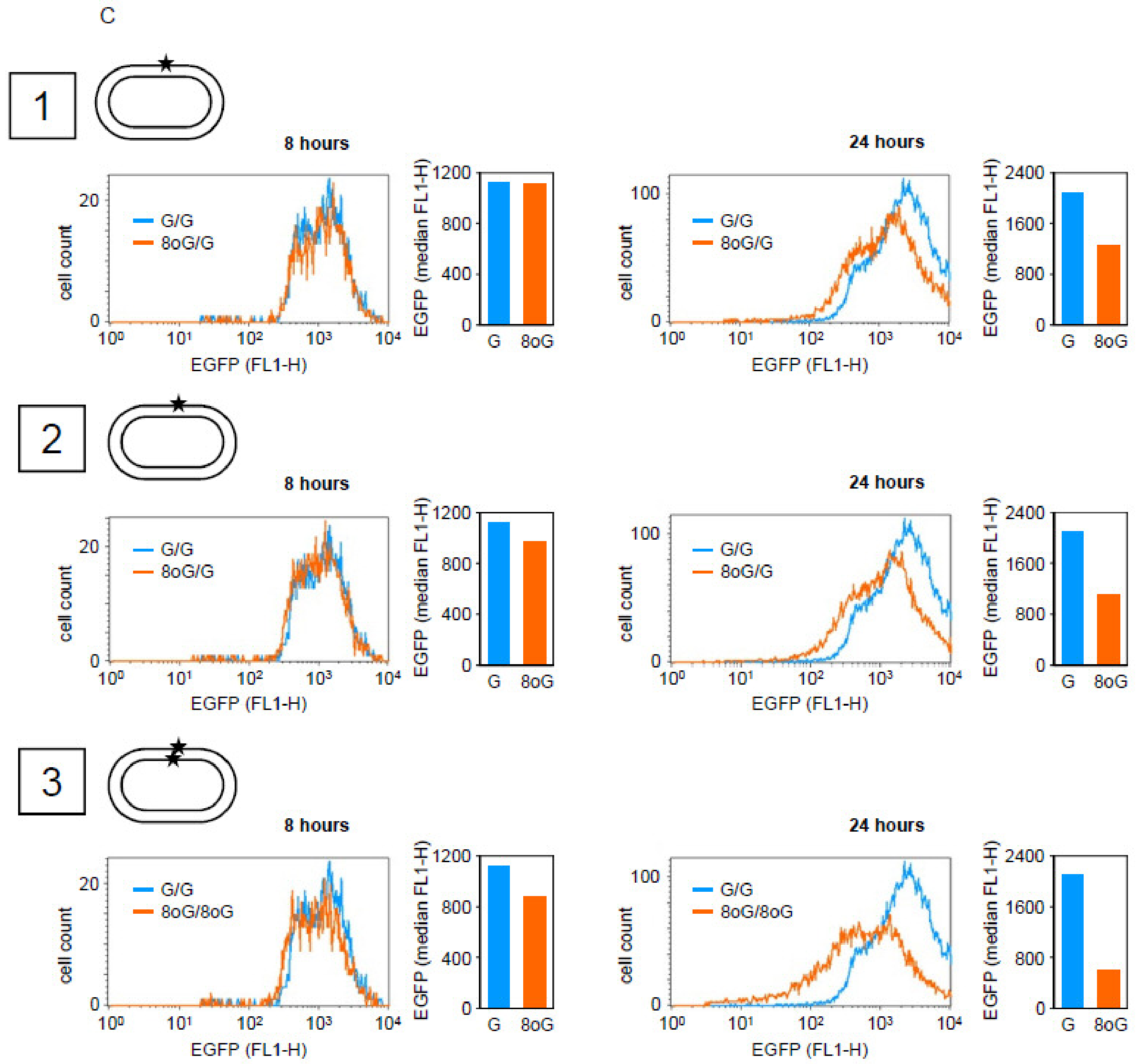

Figure S6. Construction of the clustered lesion containing simultaneously two 8-oxoG bases in the opposite DNA strands. (A) Outline of the procedure used for construction of the bistranded clustered lesion. The strand exchange procedure for individual DNA strands was described previously in great detail (31). The single-stranded DNA fragments excised by nicking endonucleases with opposite strand specificities (Nt.Bpu10I and Nb.Bpu10I) were sequentially exchanged for the matching synthetic oligonucleotides each containing single 8-oxoG. The efficiency of each strand exchange reaction was monitored by inhibition of ligation in the absence of PNK. The presence of 8-oxoG in plasmid DNA was confirmed by Fpg incision. (B) Incision activity of Fpg DNA glycosylase in the indicated plasmid substrates containing single (1, 2) or clustered (3) 8-oxoG. (C) Expression of vectors containing single (1, 2) or clustered (3) 8-oxoG in HeLa cells. Representative results obtained in one of three independent transfection experiments.

Table S1. Sequences (5′→3′) of the oligonucleotides used for targeted incorporation of 8-oxoG in varying sequence contexts. G* indicates guanine or 8-oxoG. Suitable vector/nicking enzyme pairs, positions of 8-oxoG with respect to the EGFP gene elements, and the DNA strand are specified for each oligonucleotide.

Oligonucleotide	Vector	Nicking enzyme	Gene element	DNA strand
TGAGCACCCAGTCCG*CCC	pZAJ-3w-AGC	Nt.Bpu10I	CDS	non-transcribed
TCAGGGCG*GACTGGGTGC	pZAJ-3w-AGC	Nb.Bpu10I	CDS	transcribed
CATTGCTTCGCTAG*CACG	pZAJ-5w-AGC<br>pZAJ-5c-AGC<br>pZAJ-3w-AGC<br>pZAJ-3c-AGC	Nb.BsrDI<br>Nb.BsrDI<br>Nb.BsrDI<br>Nb.BsrDI	5′-UTR<br>5′-UTR<br>3′-UTR<br>3′-UTR	non-transcribed<br>transcribed<br>non-transcribed<br>transcribed
CATTGCAGGGCG*GACTGA	pZAJ-5w-CGG<br>pZAJ-5c-CGG	Nb.BsrDI<br>Nb.BsrDI	5′-UTR<br>5′-UTR	non-transcribed<br>transcribed

SUPPLEMENTARY METHODS

Construction of vectors for site-specific incorporation of 8-oxoG

The BsrDI site of the pEGFP-mODC-ZA vector (52) was mutated by PCR with PfuTurbo DNA polymerase (Agilent, Böblingen, Germany) and overlapping primers. The procedure was based on the protocol described previously (53), with the following modifications. Primers were: 5′-CATCATGGCTGATGCTATGCGGCGGCT and 5′-AGCCGCCGCATAGCATCAGCCATGATG. The reaction products were digested with DpnI (NEB GmbH, Frankfurt am Main, Germany), cleaned up, and the PCR product was circularised by homologous recombination in *E. coli scs-8* strain. The obtained pZAJ plasmid lacking the BsrDI site was further modified as following. A DNA sequence was designed with a 5′-AGC-3′ trinucleotide amid a dodecanucleotide with an average base content flanked by two unidirectional Nb.BsrDI sites. Suitable adapters were added to achieve insertion of the same sequence in alternative orientations between the NheI and AgeI sites in the 5′-UTR of the EGFP gene. The same was done for the SalI and KpnI sites in the 3′-UTR. Vectors for the incorporation of 8-oxoG in a 5′-CGG-3′ context were designed in an analogous way. DNA sequences of the inserts are shown in Supplementary Figure S1.

Stable knockdown of the OGG1 protein expression in HeLa cells

Several candidate sequences for shRNA-targeting of the human OGG1 gene (HGNC:8125) transcripts were chosen by different algorithms. Selected sequences were reconstituted from synthetic oligonucleotides which were annealed and cloned into the pENTR/pSuper+ vector (Addgene, Cambridge, MA). The shRNA-coding sequences were inserted between the available BglII and HindIII sites for expression under the control of the H1 promoter. The obtained plasmids were co-transfected into HeLa cells with the help of Effectene (QIAGEN GmbH, Hilden, Germany) together with a pcDNA3 vector (Life Technologies GmbH, Darmstadt, Germany) containing a neomycin resistance gene in ratios 8:1. Transfected cells were selected in DMEM medium containing G418 (Life Technologies). 16 to 25 individual clones were picked for each construct and propagated under selection for at least two more weeks before analyses.

Analyses of the OGG1 protein expression in stable clones were performed by Western blotting with the EPR4664(2) anti-OGG1 rabbit monoclonal antibody (Epitomics, Burlingame, CA). The C4 monoclonal antibody (Santa Cruz Biotechnologies Inc., Heidelberg, Germany) was used to detect beta-actin as the loading reference. Several clones (all derived from transfection with the same shRNA

construct) showed a significant decrease of the OGG1 protein level. The sequences of the oligonucleotides used for cloning in this case were the following: 5′-GATCCCCGGATCAAGTATGGACACTGTTCAAGAGACAGTGTCCATACTTGATCCTTTTTA and 5′-AGCTTAAAAAGGATCAAGTATGGACACTGTCTCTTGAACAGTGTCCATACTTGATCCGGG.

Induction of oxidative base modifications by potassium bromate and the repair analyses

G418 was removed 24 hours before damage induction, and 3 $\times 10^6$ Hela cells stably expressing the OGG1 shRNA or those transfected with the empty vector were plated per flask (one 75 cm^2 flask per each mode of treatment and each repair time). $KBrO_3$ was dissolved in serum-free DMEM (PAA, Pasching, Austria) to obtain a 7.5 mM solution. Immediately prior to the damage induction, cells were washed twice with phosphate-buffered saline (PBS) at 37 °C, followed by addition of pre-warmed 10 ml DMEM or the $KBrO_3$ solution (37 °C). Flasks were placed for 15 min in an incubator for damage induction, after which cells were washed twice with PBS and either harvested directly by trypsinisation, or supplemented with full medium containing 10% FCS and returned to the incubator to allow repair for 1, 2, 4 and 6 hours. Detached cells were mixed with ice-cold DMEM, centrifuged (380 ×g, 5 min), and pellets resuspended in 2 ml DMEM containing 10% DMSO and preserved at -80 °C. DNA damage analyses were done on the next day.

The amounts of Fpg-sensitive base modifications generated by potassium bromate were quantified by the alkaline elution method for quantitative detection of single-strand breaks (SSB) in genomic DNA originally described by Kohn and co-workers (54). The assay is based on the fact that the elution rates of denatured high molecular weight DNA from polycarbonate filters are accelerated in the presence of SSB. The procedure was further extended for simultaneous detection of oxidative base modifications by introducing treatment of parallel samples with bacterial Fpg DNA-glycosylase which recognises oxidised purines and abasic sites converting them to SSB (55,56). For the chosen damaging conditions, 8-oxoG is the major lesion recognised by Fpg, while the other Fpg-sensitive base modifications are generated in many fold smaller amounts and abasic sites are not detectable (data not shown). Recombinant Fpg was purified from E. coli, as described previously (57). The alkaline elution protocol was described in every detail previously (55,56). For every mode of treatment/repair, 10^6 cells were immobilised per filter, lysed, and proteinase K-treated in triplicates. Two of three filters were treated with Fpg (3 µg/ml) to determine the sum of SSB and the Fpg-sensitive base modifications. The remaining filter was used for direct detection of SSB.

SUPPLEMENTARY REFERENCES

31. Lühnsdorf, B., Kitsera, N., Warken, D., Lingg, T., Epe, B. and Khobta, A. (2012) Generation of reporter plasmids containing defined base modifications in the DNA strand of choice. Analytical biochemistry, 425, 47-53.

52. Khobta, A., Kitsera, N., Speckmann, B. and Epe, B. (2009) 8-Oxoguanine DNA glycosylase (Ogg1) causes a transcriptional inactivation of damaged DNA in the absence of functional Cockayne syndrome B (Csb) protein. DNA repair, 8, 309-317.

53. Shareef, M.M., Dancea, H.C., Gross, J.L., Myers, T.T., Griggs, W.W., Ahmed, M.M. and Sheldon, D.G. (2008) A noncommercial polymerase chain reaction-based method to approach one hundred percent recombinant clone selection efficiency. Analytical biochemistry, 382, 75-76.

54. Kohn, K.W., Erickson, L.C., Ewig, R.A. and Friedman, C.A. (1976) Fractionation of DNA from mammalian cells by alkaline elution. Biochemistry, 15, 4629-4637.

55. Epe, B., Pflaum, M. and Boiteux, S. (1993) DNA damage induced by photosensitizers in ellular and cell-free systems. Mutation research, 299, 135-145.

56. Epe, B. and Hegler, J. (1994) Oxidative DNA damage: endonuclease fingerprinting. Methods in enzymology, 234, 122-131.

57. Boiteux, S., O'Connor, T.R., Lederer, F., Gouyette, A. and Laval, J. (1990) Homogeneous Escherichia coli FPG protein. A DNA glycosylase which excises imidazole ring-opened purines and nicks DNA at apurinic/apyrimidinic sites. The Journal of biological chemistry, 265, 3916-3922.
